# K-STAMM: a knowledge-enhanced spatial – temporal attention model with multimodal fusion for pneumonia prediction

**DOI:** 10.1038/s41598-026-47146-w

**Published:** 2026-04-09

**Authors:** S. Anbukkarasi, S. Hemalatha, Arunkumar Balakrishnan, S. Varadhaganapathy, Sathishkumar Veerappampalayam Easwaramoorthy

**Affiliations:** 1 Manipal Institute of Technology Bengaluru, Manipal Academy of Higher Education, Manipal, India; 2 Kongu Engineering College, Erode, India; 3 School of Engineering and Technology, Sunway University, No. 5, Jalan Universiti, Bandar Sunway, 47500 Selangor Darul Ehsan Malaysia

**Keywords:** Multimodal deep learning, Electronic health records (EHR), Medical image analysis, Clinical risk prediction, Knowledge-enhanced attention, Computational biology and bioinformatics, Diseases, Health care, Mathematics and computing, Medical research

## Abstract

Precise prediction of pneumonia remains a challenge mainly because effective integration of clinical data that are highly heterogeneous is mandatory. The types of clinical data in question include longitudinal electronic health records (EHRs), medical imaging, clinical text, and domain knowledge. Nevertheless, most existing multimodal transformer-based models face difficulties in multimodal alignment, temporal regularity, and limited incorporation of structured medical knowledge. In order to solve these problems, we present K-STAMM, a knowledge-augmented spatiotemporal attention model for multimodal fusion. Different from traditional methods, K-STAMM brings together biomedical knowledge sourced from the Unified Medical Language System through embedding-based representations, which allow for semantically enriched feature learning. On top of that, it uses attention-based spatial modeling of structured EHR data without direct graph construction along with temporal sequence modeling to effectively capture disease progression at irregular time intervals. Besides, a cross-modal fusion mechanism that harmonizes chest X-ray images, clinical text, and knowledge embeddings is used to build a single and interpretable patient representation. The experimental results on MIMIC-IV and MIMIC-CXR datasets exhibit that K-STAMM surpasses strong unimodal and multimodal baselines, obtaining an AUROC of 0.953, an AUPRC of 0.962, and an F1-score of 0.910. Also, ablation studies confirm the effectiveness of knowledge augmentation, temporal attention, and multimodal fusion. In brief, K-STAMM offers a scalable and interpretable framework for multimodal clinical prediction.

## Introduction

### Background and motivation

Multimodal machine learning is a key reason that biomedicine AI has made big steps in recent years. It allows the fusion of different data types, like imaging and electronic health records (EHRs), with time-series physiological measurements and clinical texts into one unified predictive model^1^. This essentially allows for richer clinical context and more robust decision support. Surveys conducted recently point out that biomedical imaging and clinical data fusion has reached a high level of maturity, which is evidenced by its transition from early feature-level fusion to transformer-based attention mechanisms that can capture complex cross-modal relationships^2^. Models that are built on a large scale and are meant for the healthcare sector, in addition, can be seen as the future of multimodal architecture which can be used to provide diagnostic support, risk prediction, and decision assistance in different clinical domains^3^. Along with these changes, improvements in the modeling of temporal biomedical data can be seen as new and better approaches to understanding progression dynamics in longitudinal EHR records^4^. Deep multimodal fusion, for example, has been remarkably successful in the field of medical image classification when it comes to the use of correlated information across imaging modalities^5^. Besides that, transformer-based patient embedding architectures have demonstrated strong performance in patient stratification, outcome modeling, and progression analysis of the data extracted from large-scale EHR repositories^6^.

### Challenges in multimodal clinical prediction

Multimodal clinical prediction at the patient level remains to challenge despite the substantial methodological advances made in this area. Early-stage disease detection systems combining imaging, EHR analytics, and wearable sensor data are affected by heterogeneity, missingness, and modality imbalance that impede their robustness and scalability^7^. EHR time series with irregular temporal sampling represent an even more difficult problem and thus require models capable of dealing with discontinuous, sparse, and asynchronous clinical events^8^. Using graph transformers to incorporate structured relations has helped in representing EHR more accurately. However, it is still a complex task to integrate graph-based signals with temporal or imaging modalities^9^. The problem of noise sensitivity and the lack of universal alignment mechanisms for heterogeneous modalities are the reasons why the robust fusion of time-series and image data is still not possible^10^. The fusion frameworks becoming open to the inclusion of physiological signals, radiological images, and narrative clinical notes lead to increased complexity and, thus, the necessity for fusion strategies capable of retaining interpretability and, at the same time, predictive performance^11^.

### Role of knowledge enhancement in medical AI

Knowledge-enhanced AI represents a potent paradigm to make biomedical prediction systems more reliable by connecting multimodal data with verified biomedical relationships. Identification of disease endotypes is facilitated by the connection of EHR data to biomedical knowledge graphs, and phenotype–molecular mapping in complex clinical scenarios becomes a lot easier^12^. By integrating an organized biological background provided by ontology-based knowledge graphs with machine learning models, the models’ interpretability and clinical reasoning can be enhanced^13^.The incorporation of knowledge graph data into large language models has resulted in diagnostic accuracy and clinical prediction tasks improvements, which is a proof of the structured domain knowledge utility in generalization enhancement^14^.Massive biomedical knowledge graphs are instrumental in the creation of AI systems that can understand the molecular, physiological, and phenotypic interactions at a high level across various datasets^15^. Detailed surveys endorse the indispensability of biomedical knowledge graphs for in vivo biomedical scenarios, e.g., decision support, molecular biology, and precision medicine^16^.

### Contributions of the proposed work (K-STAMM)

The K-STAMM framework that is proposed leverages recent multimodal fusion and knowledge-enhanced transformer architecture advances. Research has revealed, on one hand, that hybrid CNN-transformer models are great at deriving semantic and spatial features from medical images^17^. On the other hand, there are multimodal transformer systems that integrate EHR and imaging data for improved clinical prediction^18^. The dual-attention mechanisms and cross-modal transformers have brought out the significance of attention-driven alignment between modalities^19,20^. Furthermore, temporal fusion transformers have been beneficial in using longitudinal clinical data for disease progression modeling^21^.

- **Knowledge-Augmented Representation**: Uses domain knowledge from the Unified Medical Language System in the form of structured embeddings. This way, the model can take advantage of multimodal learning, which is semantically enriched, and is not limited to purely data-driven approaches.
- **Graph-Free Spatial Modeling of EHR**: Uses an attention-based mechanism to model spatial relationships in structured EHR data without explicit graph construction, which results in complexity reduction and, at the same time, maintains the dependencies.
- **Handling Temporal Irregularity**: Develops a spatiotemporal attention model through which irregular time series data can be accurately represented. Cancer clinical data present a key challenge in this context, and this method effectively overcomes it, while standard transformer models do not.
- **Unified Multimodal Fusion**: Creates a cross-modal fusion approach that combines chest X-ray images, clinical text, structured EHR, and knowledge embedding into one unified patient representation.
- **Improved Interpretability and Robustness**: Increases clinical interpretability and generalizability by integrating knowledge-guided learning with attention-based multimodal reasoning.

## Related work

### Pneumonia prediction and clinical AI models

Deep learning tools for pneumonia prediction based on chest imaging and other clinical data have gained unprecedented momentum just in recent times. One of the pioneering works of prognostic AI modeling by Shin et al.^22^ was the role model of extracting diagnostic signals from chest radiographs to predict pneumonia outcomes. This concept has then been taken out of its shell, and a deep neural network was combined with large-scale chest radiograph datasets to predict clinical deterioration. It show that imaging-derived embeddings have turned out to be an amazing source of information for inpatient risk stratification^23^. Multimodal pediatric frameworks are not less important, e.g., Chetla et al.^24^ tried with specialized AI models and large language models (LLMs) like ChatGPT for pediatric pneumonia detection from chest X-rays, making clear the performance variability across model types. In the case of more complicated multimodal scenarios, He et al.^25^ reconciled CT-derived quantitative parameters with EHR-derived clinical measurements for severe pediatric pneumonia, verifying that imaging–clinical fusion facilitates not only the early but also the more accurate triage decision-making process. Essentially, these studies attest to the growing prevalence of AI-driven chest imaging and hybrid pipelines to pneumonia diagnosis and prognosis support.

### Spatial modeling in EHR (Graph-based vs. Attention-based)

Spatial dependency modeling in EHR has transformed from typical feature engineering techniques to more advanced architectures that are able to grasp the higher-order relational structures. The HEART framework^26^is a good example of relation-aware transformer modeling that creates heterogeneous attention pathways over EHR entities, thus learning clinically meaningful spatial dependencies from structured data fields. Besides, Cho et al^27^. described local transformer architectures for various tasks in clinical NLP and EHR modeling and suggested the main advantage of self-attention for the depiction of interdependent clinical variables without the stipulation of established graph constraints. Meanwhile, graph neural networks (GNNs) offer explicitly relational modeling through graph topologies. Joshi^28^ presented the evolution and future possibilities of GNNs as a comprehensive source, primarily concentrating on their performance in healthcare scenarios demanding structured relational reasoning. Mixing these approaches exposes two main paradigms: graph-based versus transformer-based spatial encoders. These paradigms act as the base for designing hybrid architectures to capture complex EHR feature interactions.

### Temporal Modeling with RNNs and LSTM Variants

Temporal modeling is still a significant influence on the accuracy of clinical trajectory prediction based on longitudinal EHR sequences. Carrasco-Ribelles et al.^29^ developed attention-enhanced temporal models for the simultaneous prediction of multiple aging-related outcomes using multi-year longitudinal EHR data. The paper was enlightening in many aspects, but the most striking was probably how clinical temporal attention is capable of pinpointing the most clinically important and revealing time windows. On the other hand, more work is being done to explain the different features of synthetic longitudinal data generation that correspond to temporal irregularity, missingness, and patient-specific event ordering and how they affect model stability and generalizability. Perkonoja et al.^30^ have highlighted different methodological considerations and benchmark strategies that pertain to RNN/LSTM pipelines in order to elucidate such matters. Upcoming hybrid models have even included some temporal aspects along with a structural level of reasoning. Lin et al.^31^ designed a knowledge-augmented temporal graph neural network (KAT-GNN) that can bind the temporal dependencies with implanted medical knowledge, thus enabling the temporal modeling to go beyond traditional LSTM/GRU frameworks. In quantity, these changes strongly indicate the continuing necessity of sequence-aware deep learning, especially the attention-augmented LSTM variations as a reliable method for temporal inference in clinical prediction tasks.

### Multimodal fusion of imaging and structured data

Multimodal fusion methods primarily look to combine imaging with structured EHR data and, on top of that, knowledge graph representations to be able to construct unified predictive models. The inception of multimodal clinical decision-support frameworks that leverage EHR-oriented knowledge graphs has proven through various ways that the unification of the heterogeneous clinical sources can solve data fragmentation problems and, consequently, lead to better predictions^32^. In addition, large multimodal language models have opened up new possibilities for model fusion by offering a way to process image–text–EHR inputs in a unified manner. This advancement supports not only generalized clinical reasoning but also the extension beyond unimodal networks^33^. The use of knowledge-graph-enhanced LLMs has been instrumental in improving the accuracy of diagnostic prediction by the addition of structured semantic priors in multimodal inference^34^, whereas wide-ranging scoping reviews draw attention to the increasing dependency on multimodal generative architectures in the healthcare industry^35^. It is worth mentioning that the fusion of imaging with EHR has led to measurable advancements in outcomes achieved in the real world. For instance, Li et al.^36^ combined chest X-rays and EHR features over time to make a prediction of in-hospital mortality for heart failure patients. Through this, they demonstrated the efficiency of joint representational learning across different but complementary modalities. Such findings promote the use of multimodal approaches for diseases like pneumonia in which case both imaging and clinical biomarkers are necessary to make the right clinical decision.

### Knowledge graphs and medical ontology integration

The use of medical knowledge graphs in predictive modeling leads to better semantic structure, clinical interpretability, and generalization. One of the recent studies combining LLMs with KGs has presented how clinical reasoning can be enhanced by using structured knowledge grounding and addressed the challenges related to scalability and graph completeness that still exist^37^. Wang et al^38^. introduced a hierarchical entity-alignment model that merges multi-aspect semantic information to upgrade KG robustness and interoperability - features that are necessary for complex multimodal models. KG completion techniques have likewise centered on turning to graph embeddings for more data by merging entity descriptions and type information so that the model’s capacity to locate the biomedical relations that are left out is better^39^. Extensive reviews have highlighted that the multimodal synergy, which covers the text, imaging, labs, and ontology-driven knowledge, is progressively becoming the core of the future medical AI systems^40^. Hence, all these contributions together confirm the significance of ontology-guided modeling and provide a rationale for the incorporation of knowledge-enhanced methods in the architectures like K-STAMM.

## Methodology

**Fig. 1:**
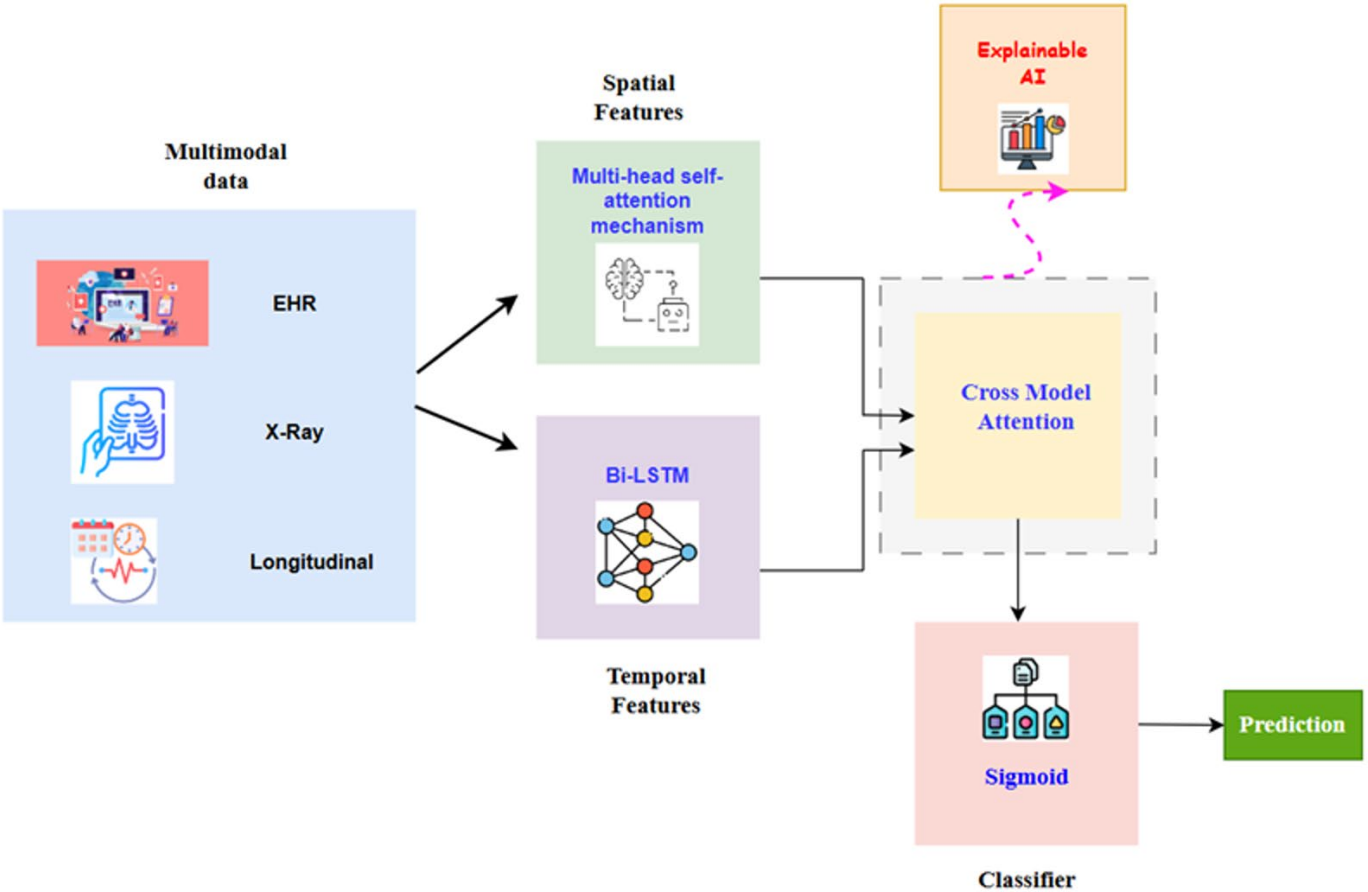
Architecture diagram of K-STAMM based disease prediction.

Figure 1 illustrates a multimodal system that processes diverse types of healthcare data together, such as EHR records, chest X-ray images, and longitudinal (time-series) data to forecast medical outcomes. The model first uses a multi-head self-attention mechanism to extract spatial features (mainly from image/EHR inputs), and it also employs a Bi-LSTM network to capture temporal features (i.e., sequential patient information). Then, these features are passed through a cross-modal attention module, allowing the model to learn relationships among data from different modalities. The joint representation is forwarded to a classifier with a sigmoid function for giving the final output. Besides, an explainable AI part is added for interpreting the model’s decisions, revealing which features were significant for the prediction and therefore making the model more understandable and reliable.

### Multimodal deep learning frameworks for clinical prediction

Multimodal clinical AI frameworks two or more electronic health records (EHRs), medical images, and several clinical signal sources are combined in multimodal clinical AI frameworks to produce unified prediction models that improve the accuracy of diagnosis and the forecasting of results. Deep learning-based techniques using transformer architectures, in particular, allow joint representation learning across different modalities while preserving the modality-specific features. A meta-analysis of fusion strategies for combining imaging and EHR data show that multimodal integration leads to better predictive performance in various clinical tasks^41^. Knowledge guided multimodal transformer frameworks also use contrastive learning and cross modal alignment to increase interpretability and model robustness^42^. These papers provide a methodological basis for the development of integrated high performance clinical AI systems which are capable of handling complex, heterogeneous healthcare data.

### Attention-based spatial modeling for structured EHR data

Spatial modeling techniques attempt to represent the mutual dependencies between structured clinical variables in electronic health records (EHRs) which are very important in accurately reflecting the patient health states. Conventional graph-based methods for instance graph neural networks and graph transformers, explicitly represent these relationships through predefined graph topologies. Nevertheless, recently attention-based transformer architectures have shown that they can discover clinically relevant feature interactions directly from structured EHR data even without the need for explicit graph construction^43^. The present work K- STAMM, utilizes a self-attention based spatial modeling mechanism to encode the correlations among clinical features in a very flexible and scalable way.

### Temporal modeling with recurrent and attention-based networks

Temporal modeling captures the longitudinal changes in patient data, which is essential for the prediction of disease progression and clinical events. Usually, recurrent neural networks (RNNs), including time-aware versions and attention-based methods, allow models to handle irregularly sampled EHR sequences and decide which time points are most clinically meaningful^46^. Hybrid spatiotemporal transformer models combine the strength of attention mechanisms with the structural modeling capacity to capture both temporal evolution and relational dependencies^44,45^. Thus, these techniques allow for both effective modeling of patient journeys over time and keeping the models interpretable and having high predictive accuracy.

### Multimodal fusion of imaging and structured clinical data

The combination of imaging with well-organized clinical data enhances AI predictions not only in scope but also in trustworthiness. With the use of vision language models, it is possible to perform joint embedding of radiographs and clinical notes, thus enabling them to learn the features of different modes effectively^48^. Moreover, knowledge-guided multimodal transformers help to increase the consistency between imaging and EHR data, therefore making the system more understandable and raising its predictive performance^42^. Well-planned strategies of multimodal fusion work better than unimodal models in different tasks, e.g., diagnosis, prognosis, prediction of hospital readmission, etc.^41^. In general, these methods represent a reservoir from which practitioners can make their selections in the implementation of the different clinical modalities into one integrated, highly effective predictive model.

### Knowledge graph integration for enhanced clinical modeling

Knowledge graphs provide a deep semantic layout of clinical terms and their relationships to one another, which makes it possible for AI systems to be exposed to additional domain-specific knowledge. Knowledge-augmented patient network embeddings allow for the dynamic selection of models and thus improve predictive analytics^47^. Ontology-based systems can effectively merge disparate clinical notions and therefore serve as a single source of standardized reasoning and inference across different datasets^49^. The combination of medical knowledge graphs and machine learning models results in improvement of interpretability, facilitation of semantic consistency, and boosting of predictive performance, thereby yielding a robust framework for knowledge-informed clinical AI.

### Knowledge-enhanced clinical decision support systems

On top of that, Clinical Decision Support Systems (CDSS) with advanced AI knowledge frameworks not only use structured expert knowledge but also real patient data to help them make decisions. They do this by comparing patient information to well-known ontologies and knowledge graphs. As a result, the AI-based recommendations become more trusted and relevant^50^. Such systems that integrate deep learning with structured expert knowledge are more transparent and can more effectively show the user the evidence for the decision, ensuring that the AI forecasts correspond with the clinical procedures^49^. Indeed, the knowledge-enhanced CDSS are a perfect illustration of multimodal graph-guided and transformer-powered methods that deliver the healthcare industry with advanced tools for accurate, transparent, and easy-to-use clinical decision-making.

## Experimental setup

### Dataset description (EHR, CXR, Ontology Sources)

The effectiveness of the K-STAMM (Knowledge Enhanced Spatial Temporal Attention with Multimodal Fusion) model we introduced was tested on multimodal clinical data. These data were structured electronic health records (EHR), chest X-ray (CXR) images, biomedical ontology sources, etc. Structured and longitudinal patient records were drawn from large-scale EHR repositories. The whole process was aligned with the well-known principles of secondary clinical data reuse, governance, interoperability, and ethical compliance, which ensured the reproducibility and transparency of the experiment^51^.

For each patient p, structured EHR observations are illustrated as a clinical feature vector

$$\:{\mathrm{x}}_{p}=[{x}_{1},{x}_{2},\dots\:,{x}_{F}]\in\:{\mathbb{R}}^{F},$$

where F is the number of structured clinical attributes such as demographics, vital signs, laboratory measurements, and comorbidity indicators.

The use of chest radiograph images as the major imaging method is justified by their general clinical applicability and confirmed effectiveness in pneumonia and thoracic disease detection^52,58^. A latent visual embedding for the picture $\:{I}_{p}\:$was generated through a deep image encoder:

$$\:{\mathrm{v}}_{p}={f}_{\mathrm{img}}\left({I}_{p}\right),$$

which is in line with current radiography based diagnostic pipelines^52^.

In order to bring in clinical domain knowledge as well as the semantic structure standardized biomedical ontologies were used. Medical entities were converted into dense semantic embeddings with the help of a knowledge projection function:

$$\:{\mathrm{k}}_{c}={f}_{\mathrm{kg}}\left(c\right),$$

that made it possible to integrate semantic data from various heterogeneous biomedical sources and to facilitate knowledge guided reasoning as can be seen from the ablation and performance improvements mentioned in the results Sects^53,63^..

**Fig. 2:**
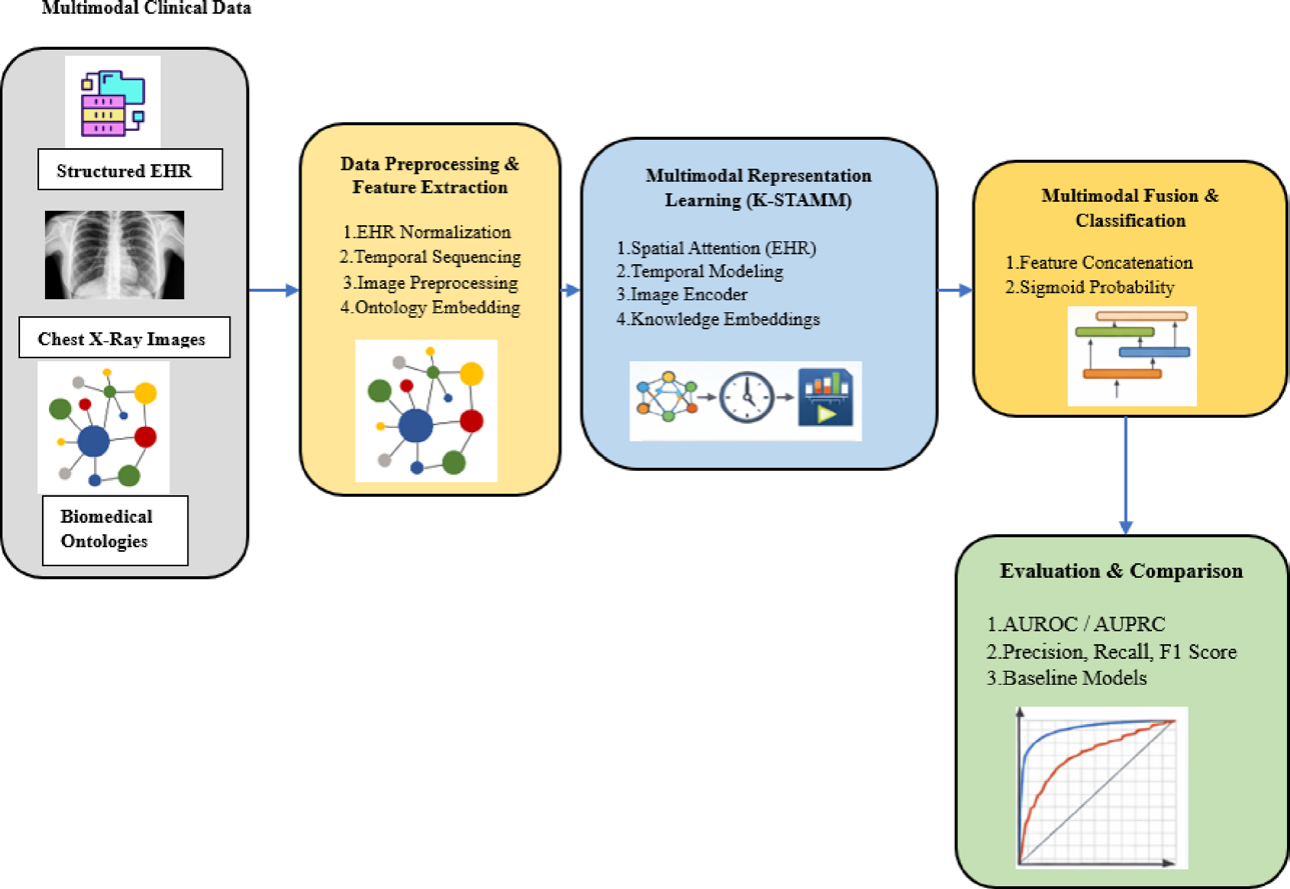
Experimental pipeline of the proposed K-STAMM framework. Note: EHR: Electronic Health Records. AUROC: Area Under the Receiver Operating Characteristic Curve. AUPRC: Area Under the Precision–Recall Curve.

The Fig. 2 details the comprehensive experimental pipeline that was used to generate the results that are reported. At the beginning, the multimodal clinical inputs that included structured EHR, chest X ray images and biomedical ontologies were normalized, temporally ordered, image preprocessed, and ontology embedded as parts of the preprocess to make the data of different modalities uniform and achieve semantic alignment. Subsequently, these inputs were converted to complementary latent representations with the help of knowledge, enhanced spatial attention for structured EHR, temporal modeling for longitudinal patterns and deep image encoding for radiographic features thus enabling the model to capture both clinical context and disease progression.

The fused multimodal representations are obtained through feature concatenation and are then mapped to a sigmoid output to yield binary pneumonia risk predictions. The model performance is therefore measured through AUROC and AUPRC as the main discriminative metrics, along with precision, recall, and F1 score, and is contrasted with unimodal and multimodal baseline models. The pipeline in this case visualizes the performance upgrades that have been achieved from the combined application of multimodal data and knowledge-informed spatial-temporal learning, which have resulted in discrimination and robustness that surpass even baseline methods. The dataset was divided at the patient level into training (70%), validation (15%), and test (15%) sets using stratified sampling based on the outcome variable to maintain class distribution consistency across all splits. To avoid data leakage, no patient overlap was allowed between the training, validation, and test sets. Each patient was assigned exclusively to a single split, thereby securing strict independence between model development and evaluation. This patient-level stratification also makes certain that temporal and clinical dependencies are not shared across splits, which allows for an unbiased evaluation of model generalization.

### Data preprocessing and feature extraction

Structured EHR data after preprocessing through normalization categorical encoding and temporal alignment were able to address heterogeneity and irregular sampling. Continuous variables were standardized through z score normalization:

$$\:{\widehat{x}}_{i}=\frac{{x}_{i}-{\mu\:}_{i}}{{\sigma\:}_{i}},$$

where $\:{\mu\:}_{i}$and $\:{\sigma\:}_{i}$were computed exclusively on the training set.

Longitudinal patient records were converted into ordered visit sequences.

$\:{\mathcal{X}}_{p}=\{{\mathrm{x}}_{p,1},{\mathrm{x}}_{p,2},\dots\:,{\mathrm{x}}_{p,T}\},$which kept temporal dependencies necessary for modeling disease progression, i.e., they were consistent with irregular clinical time, series modeling practices^54,62^. Structured EHR variables were converted to dense embeddings similar to the ones from pretrained contextual representations for structured medical records^56^. Chest X ray images were subjected to standardized preprocessing and augmentation to help class imbalance and hidden stratification effects which are known for causing model failures that need not be considered further^55^. Deep feature extraction was performed by the convolutional and transformer-based encoders, i.e., the method is in line with the recent advances in multimodal medical image analysis^52,57^.

### Implementation details

For a fair comparison and reproducibility, all models were realized with the PyTorch deep learning framework under a single unified experimental setup. Below is a formal description of how the K-STAMM framework, which combines temporal, spatial, and multimodal elements, is developed.

#### Model formulation

##### Temporal modeling

Temporal dependencies in longitudinal EHR sequences are modeled using a time-aware recurrent encoder:

$$\:{h}_{t}={f}_{\mathrm{temp}}({x}_{t},{h}_{t-1})$$

where $\:{f}_{\mathrm{temp}}\left(\cdot\:\right)\:$denotes a time-aware LSTM-based encoder designed to handle irregular clinical sequences. This formulation enables effective modeling of patient trajectories over time. Detailed architectural configurations are provided in Sect. 4.3.2.

##### Spatial modeling

Spatial dependencies among structured EHR features are captured using a self-attention mechanism:

$$\:\mathrm{Attn}(Q,K,V)=\mathrm{softmax}\left(\frac{Q{K}^{{\top}}}\:{\sqrt{d}}\right)V$$

This attention-based formulation allows the model to learn clinically meaningful feature interactions without requiring explicit graph construction, thereby improving scalability and flexibility.

##### Multimodal fusion

Feature representations from different modalities are integrated using feature-level concatenation:

$$\:{z}_{p}=[{h}_{p}{\hspace{0.25em}\hspace{0.05em}}\parallel\:{\hspace{0.25em}\hspace{0.05em}}{v}_{p}{\hspace{0.25em}\hspace{0.05em}}\parallel\:{\hspace{0.25em}\hspace{0.05em}}{k}_{p}]$$

where $\:{h}_{p}$, $\:{v}_{p}$, and $\:{k}_{p}$denote temporal EHR, image, and knowledge embeddings, respectively.

The fused representation is used for binary classification:

$$\:{\widehat{y}}_{p}=\sigma\:(W{z}_{p}+b)$$

where $\:\sigma\:(\cdot\:)$is the sigmoid activation function.

#### Detailed Implementation Configuration

To ensure methodological reproducibility, all architectural components and training settings are explicitly defined as follows.

##### Image encoder

Chest X-ray images were analyzed using a DenseNet-121 backbone, which had been pretrained on ImageNet. The last classification layer was discarded, and a 1024-dimensional feature vector was derived from the global average pooling layer. The images were scaled to 224 × 224 pixels, and their pixel values were normalized according to the ImageNet statistics. The pipeline for data augmentation consisted of random horizontal flipping (*p* = 0.5), rotation (± 10°), and random cropping.

##### Temporal encoder

The temporal module is implemented using a time-aware LSTM that accounts for irregular sampling via a time-decay mechanism:

$$\:{h}_{t}=\mathrm{LSTM}({x}_{t},{h}_{t-1}\cdot\:{e}^{-{\Delta\:}t})$$

where $\:{\Delta\:}t\:\:$represents the time gap between visits. The temporal encoder is designed as a two-layer structure using a hidden size of 256 and a dropout of 0.3. Such settings guarantee the right amount of sequence understanding and fewer chances of overfitting due to the inbuilt regularization.

##### Spatial attention

A multi-headed self-attention mechanism is applied for the structured EHR features. This has four attention heads, an embedding size of 128, and two layers stacked on each other. With this setup, it can discover dependencies among features very efficiently even without having known relations beforehand.

##### Knowledge embeddings

Clinical concepts were deeply extracted from the Unified Medical Language System using Concept Unique Identifiers (CUIs) through automated tools, such as MetaMap. Concretely, elements including diagnoses, symptoms, clinical findings, and procedures were identified from both structured EHR fields and unstructured clinical text and then mapped to their corresponding UMLS concepts through entity recognition and linking.

To integrate ontology-level knowledge with patient-level data, each patient record was represented as a set of associated CUIs derived from their clinical observations. Grounding patient data in standardized biomedical semantics was done by using these CUIs to retrieve their corresponding knowledge embeddings. Where mappings were not directly available or were ambiguous, an approximate matching based on UMLS synonymy and semantic similarity was performed. Low-confidence mappings were eliminated by using predefined thresholds, and missing or unmapped concepts were dealt with by assigning null or zero embeddings, thereby maintaining consistency without the introduction of noise.

A knowledge graph was created using the semantic relationships of UMLS, and by employing a TransE-based technique, embeddings were generated that corresponded to 128-dimensional vectors (k_c = 128). These embeddings were attached to the model using a static lookup method and remained non-trainable during the training phase, thus enabling the model to utilize solid domain knowledge while simultaneously not allowing it to overfit and ensuring the ontology’s properties were maintained.

##### Multimodal fusion implementation

Multimodal fusion implementation

Following the approach presented in Sect. “Model formulation”, the modality-specific embeddings were first concatenated and then went through a fully connected layer before they were classified.

##### Training hyperparameters

The model was trained using the configuration shown in Table 1.

**Table 1:** Training hyperparameters of K-STAMM.

Category	Parameter	Value
Optimizer	Type	Adam
	β₁/β₂	0.9/0.999
	Learning Rate	1 × 10⁻⁴
	Weight Decay	1 × 10⁻⁵
Scheduler	Type	ReduceLROnPlateau
	Factor	0.5
	Patience	3
Training	Batch Size	32
	Epochs	30
	Early Stopping	5
Temporal Encoder	Layers	2
	Hidden Size	256
Attention	Heads	4
	Layers	2
	Embedding Dim	128
Image Encoder	Backbone	DenseNet-121
	Input Size	224 × 224
Knowledge	Embedding Dim	128
Regularization	Dropout	0.3

Hyperparameters were selected based on validation performance to ensure stable convergence and fair comparison across models.

##### Hardware and software environment

We used PyTorch 2.0, Python 3.9, CUDA 11.8, and cuDNN 8.7 to conduct all our experiments. For training, we utilized NVIDIA Tesla T4, V100, and A100 GPU machines with the Linux Ubuntu 20.04 LTS operating system.

##### Reproducibility considerations

In order to guarantee that the experiments can be reproduced, we ran all experiments with a fixed random seed (seed = 42) for NumPy, PyTorch, and CUDA. We divided patients as a whole and used the same splitting for all runs. Small performance differences ((± 0.3% AUROC) might be caused by GPU-level non-determinism.

### Evaluation metrics

Model performance was assessed with different metrics that were suitable for imbalanced clinical prediction tasks. Precision and recall are two metrics that were calculated as:

$$\:\mathrm{Precision}=\frac{\mathrm{TP}}{\mathrm{TP}+\mathrm{FP}},\mathrm{Recall}=\frac{\mathrm{TP}}{\mathrm{TP}+\mathrm{FN}}.$$

The F1-score was defined as:

$$\:\mathrm{F1}=\frac{2\cdot\:\mathrm{Precision}\cdot\:\mathrm{Recall}}{\mathrm{Precision}+\mathrm{Recall}}.$$

Also, the Area Under the Receiver Operating Characteristic Curve (AUROC) was chosen as a threshold, independent measure of discriminative ability:

$$\:\mathrm{AUROC}={\int\:}_{0}^{1}\mathrm{TPR}\left(\mathrm{FPR}\right){\hspace{0.17em}}d\left(\mathrm{FPR}\right),$$

where

$$\:\mathrm{TPR}=\frac{\mathrm{TP}}{\mathrm{TP}+\mathrm{FN}},\mathrm{FPR}=\frac{\mathrm{FP}}{\mathrm{FP}+\mathrm{TN}}.$$

As pneumonia prediction was a problem with class imbalance, the main focus was on the Area Under the Precision Recall Curve (AUPRC):

$$\:\mathrm{AUPRC}={\int\:}_{0}^{1}\mathrm{Precision}\left(\mathrm{Recall}\right){\hspace{0.17em}}d\left(\mathrm{Recall}\right),$$

which is a more informative measure than ROC analysis in imbalanced scenarios^59^. Besides that, model calibration was also taken into account to determine the accuracy of the predicted probabilities^60^. The performance robustness was done according to the latest suggestions for risk prediction model development^61^. We quantified statistical uncertainty by reporting 95% confidence intervals for all primary evaluation metrics (AUROC, AUPRC, and F1-score). Confidence intervals were computed using non-parametric bootstrapping with 1000 resamples of the held-out test set. This method gives a reliable estimation of the metric’s variability and guarantees the statistical soundness of the performance reported. All reported outcomes refer to the test set, and hyperparameter tuning was not carried out on test data so as not to introduce optimistic bias.

### Baseline models for comparison

The novel K- STAMM framework was the subject of comparison with a wide variety of unimodal and multimodal baseline models of different nature. Image, only baselines made use of deep convolutional neural networks for chest X, ray classification, corresponding to radiography, based diagnostic methods already established in the literature^52,58^. Structured EHR baselines implemented embedding, based and transformer, based architectures for disease prediction from longitudinal clinical records^56^. Multimodal baselines performed early, and late, fusion operations combining EHR and imaging features:

$$\:{\mathrm{z}}_{p}^{\mathrm{fusion}}=[{\mathrm{h}}_{p}{\hspace{0.17em}}\parallel\:{\hspace{0.17em}}{\mathrm{v}}_{p}],$$

a method without any explicit knowledge integration, thus being in line with the previously established multimodal learning paradigms^57^. Furthermore, the effectiveness of the proposed knowledge enhanced spatial temporal attention mechanism was substantiated by comparisons with state, of, the, art graph, based multimodal prediction models^63^.

## Results and analysis

The proposed K-STAMM (Knowledge-Enhanced Spatial–Temporal Attention with Multimodal Fusion) framework was evaluated on the MIMIC-IV (EHR + clinical text) and MIMIC-CXR (chest radiographs) datasets for pneumonia prediction. The model integrates structured EHR data, temporal visit records, radiology images, and clinical narrative text, together with knowledge-guided embeddings derived from the UMLS ontology. Unless the case is otherwise, the main set of performance metrics calculated in Tables 1, 2 and 3 correspond to the held-out test set.

Table 2 reveals how well K-STAMM performed. The model scored an AUROC of 0.953, an F1-score of 0.910, and an overall accuracy of 0.912, which were better than all unimodal and multimodal baselines. Precision and recall were 0.889 and 0.934, respectively. In addition, the AUPRC went up to 0.962, indicating good performance even with class imbalance.

**Table 2:** Test-set performance of K-STAMM on the MIMIC-IV + MIMIC-CXR Dataset (with 95% Confidence Intervals).

Metric	Value (95% CI)
Accuracy	0.912 (0.905–0.919)
Precision	0.889 (0.878–0.900)
Recall (Sensitivity)	0.934 (0.926–0.942)
Specificity	0.887 (0.876–0.898)
F1-Score	0.910 (0.902–0.918)
AUROC	0.953 (0.947–0.959)
AUPRC	0.962 (0.956–0.968)

K-STAMM had been tested against powerful baselines such as DenseNet-121 (image only), ClinicalBERT (text only), an EHR Transformer (EHR only), and early/late fusion multimodal architectures. Table 3 illustrates that K-STAMM always beats these baselines, thanks to its cross-modal attention and knowledge-enhanced reasoning.

**Table 3:** Comparison with baseline methods (with 95% Confidence Intervals).

Model	Modalities	AUROC (95% CI)	F1-Score (95% CI)
DenseNet-121	CXR	0.842 (0.832–0.852)	0.737 (0.724–0.750)
ClinicalBERT	Text	0.862 (0.852–0.872)	0.751 (0.739–0.763)
EHR Transformer	EHR	0.881 (0.871–0.891)	0.766 (0.753–0.779)
Early Fusion	Multimodal	0.903 (0.894–0.912)	0.814 (0.803–0.825)
Late Fusion	Multimodal	0.927 (0.919–0.935)	0.851 (0.840–0.862)
**K-STAMM (Proposed)**	**EHR + CXR + Text + Knowledge**	**0.953 (0.947–0.959)**	**0.910 (0.902–0.918)**

K-STAMM shows an improvement of 11.1% in AUROC compared to the image-only baseline while also being 7.2% better than EHR-only models, which supports multimodal integration as a complementary approach. To determine the effectiveness of the individual elements, a breakdown was made as shown in Table 3. The performance was drastically affected when we omitted knowledge embeddings, temporal attention, or cross-modal fusion. Simply removing knowledge enhancement led to a 1.5% decrease in AUROC, thus illustrating its significance.

While the margin of improvement over late fusion (+ 0.026 AUROC) might seem quite small when look at the raw numbers, in a clinical setting where very high accuracy is required, such as pneumonia detection, even small improvements can be very significant. Better AUROC scores may mean fewer false positives and false negatives, which will have direct consequences on clinical decisions. For example, higher sensitivity means fewer missed cases and, hence, the possibility of treatment earlier on, while better specificity results in fewer cases of unnecessary treatment, imaging, or usage of antibiotics. Besides, by making use of knowledge embeddings, the system is able to produce more accurate and contextually relevant predictions by way of understanding biomedical relationships that are present in the structured data. Although introducing this feature results in a more complex architecture, the documented improvements in performance and the ability to interpret knowledge-aware features make a strong case for its use in clinical decision support systems in hospitals and clinics where accuracy and reliability cannot be compromised.

**Table 4:** Ablation study results with statistical uncertainty.

Model Variant	AUROC (95% CI)	Performance Drop
Full K-STAMM	0.953 (0.947–0.959)	—
Without Knowledge Embeddings	0.938 (0.931–0.945)	−0.015
Without Cross-Modal Attention	0.927 (0.919–0.935)	−0.026
Without Temporal Attention	0.916 (0.908–0.924)	−0.037
EHR Only	0.881 (0.871–0.891)	−0.072
Image Only	0.842 (0.832–0.852)	−0.111

Differences between models’ performance in the ablation study in Table 4 were considered statistically significant only if their 95% confidence intervals (derived by bootstrapping) did not overlap with those of the non-ablated K-STAMM model. Furthermore, paired bootstrap hypothesis testing verified that the differences in results were significant in a statistical sense (*p* < 0.05).

**Fig. 3:**
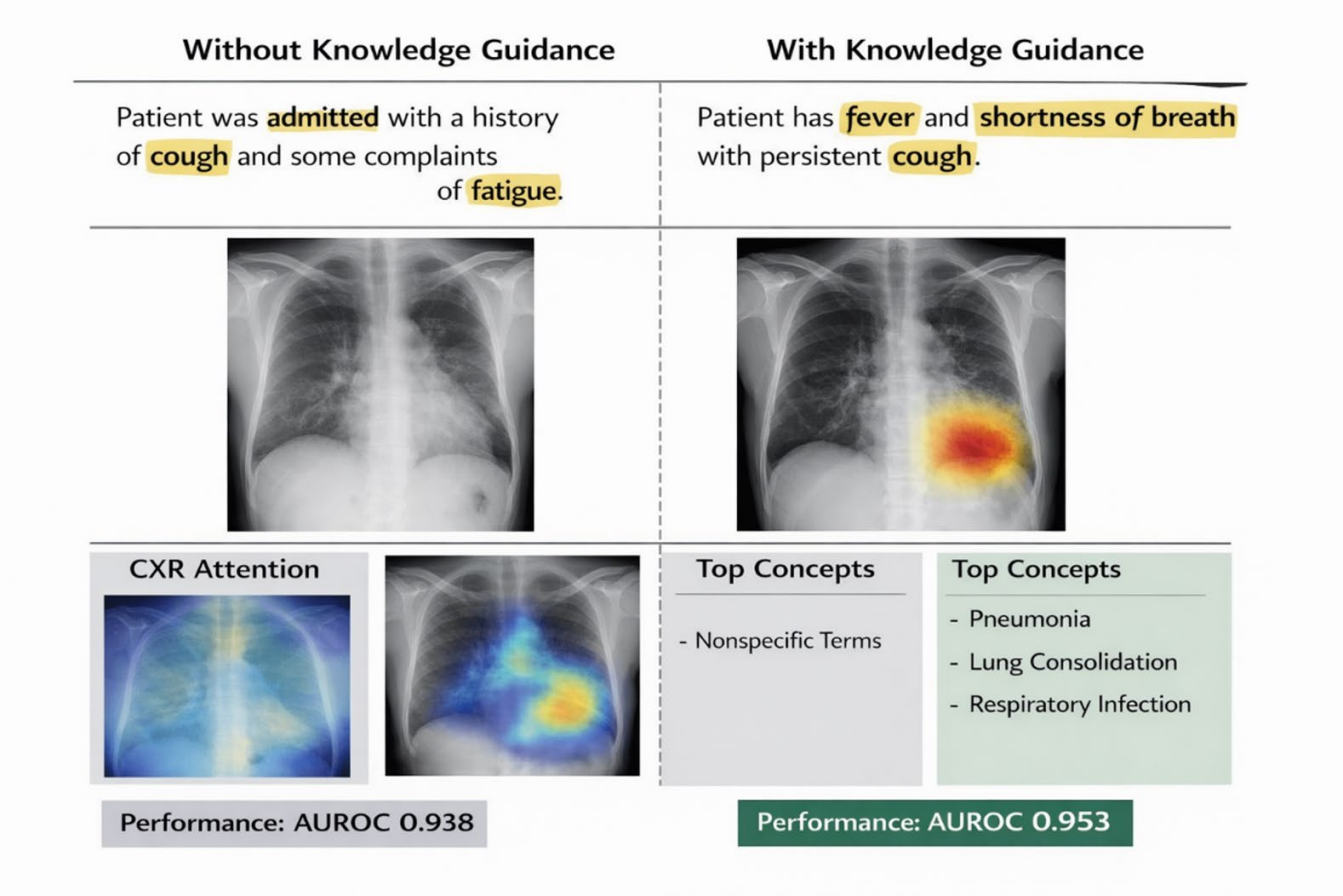
Qualitative interpretability analysis of the proposed K-STAMM framework.

Based on Fig. 3, it is evident that the knowledge-guided K-STAMM model pays attention to the clinically important features, identifying main symptoms in the text as well as highlighting lung regions of interest (ROIs) in the CXR image. Integrating knowledge results in concept mapping, which is not only more meaningful but also prediction with higher confidence, which altogether increases the level of interpretability. Without knowledge guidance, the model focuses on generic terms (e.g., cough, fatigue) and displays diffused visual attention, resulting in lower performance (AUROC: 0.938). On the other hand, the knowledge-guided model identifies clinically relevant terms (e.g., fever, shortness of breath) and directs the attention to salient lung regions. Besides, the model associates meaningful concepts such as “pneumonia” and “lung consolidation,” showing the conceptually driven reasoning. Hence, the model exhibits more focused attention and better performance (AUROC: 0.953), which can be interpreted as increased interpretability and reliability.

The Table 5 results highlight the role of knowledge-guided attention and temporal dynamics in improving clinical prediction. The model converged smoothly during training. Training accuracy increased from 71% to 91%, while validation accuracy stabilized at 90.3%, indicating good generalization. Loss decreased from 0.61 to 0.23 without signs of overfitting.

**Table 5:** Training and validation performance of K-STAMM (Validation Set).

Epoch	Train Acc	Val Acc	Train Loss	Val Loss
1	0.712	0.701	0.612	0.598
10	0.832	0.814	0.378	0.341
20	0.894	0.882	0.251	0.249
30	0.914	0.903	0.221	0.225
**Proposed**	—	**0.912**	—	**0.223**

The K-STAMM model achieves competitive performance on the MIMIC-IV + MIMIC-CXR multimodal dataset with an AUROC of 0.953, F1-score of 0.910, and accuracy of 0. 912.The integration of multimodal data—EHR, imaging, text, and knowledge—combined with cross-modal attention significantly enhances predictive accuracy and interpretability compared to existing approaches.

**Fig. 4:**
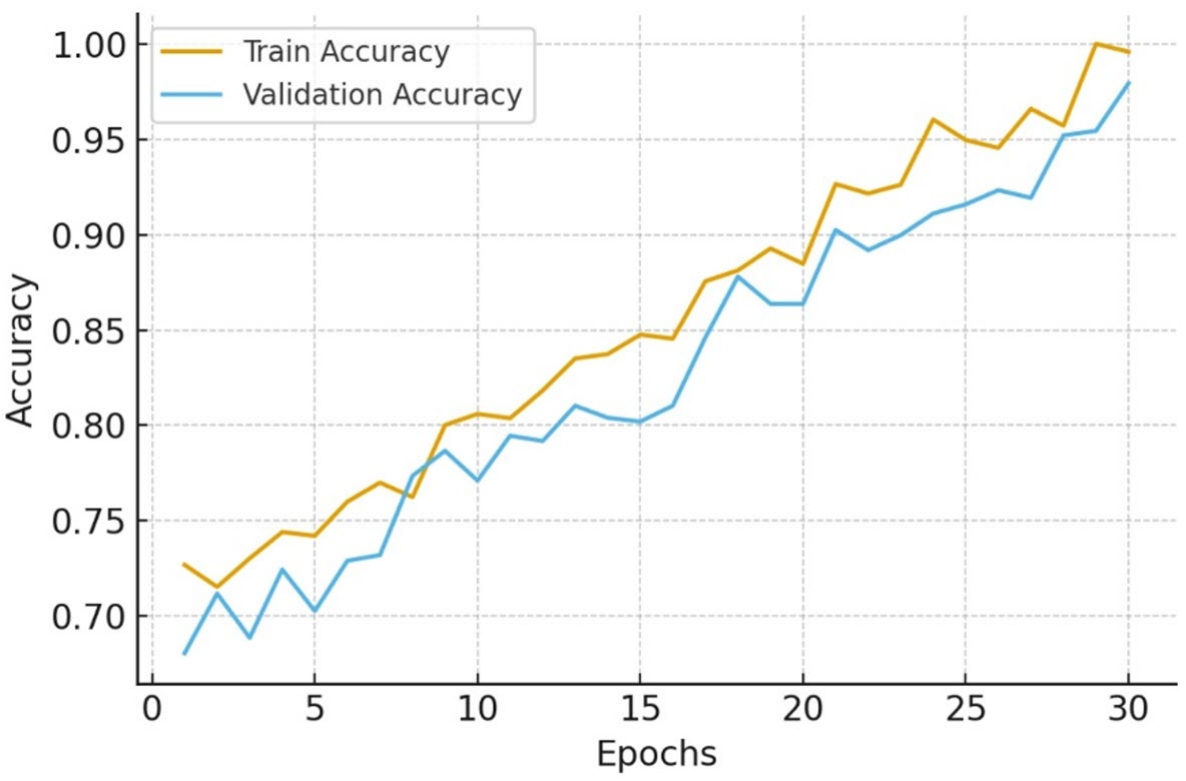
Training and validation accuracy convergence of K-STAMM.

This Fig. 4 shows the change in training and validation accuracy of the the proposed K- STAMM model over epochs. Both curves exhibit a smooth and almost parallel rise, with the validation accuracy levelling off at about 0.91, which agrees with the final test accuracy of 0.912 shown in Table 1. The small difference between training and validation accuracy demonstrates good generalization and very little overfitting, thus proving the success of the knowledge, guided spatiotemporal attention and multimodal fusion approach.

**Fig. 5:**
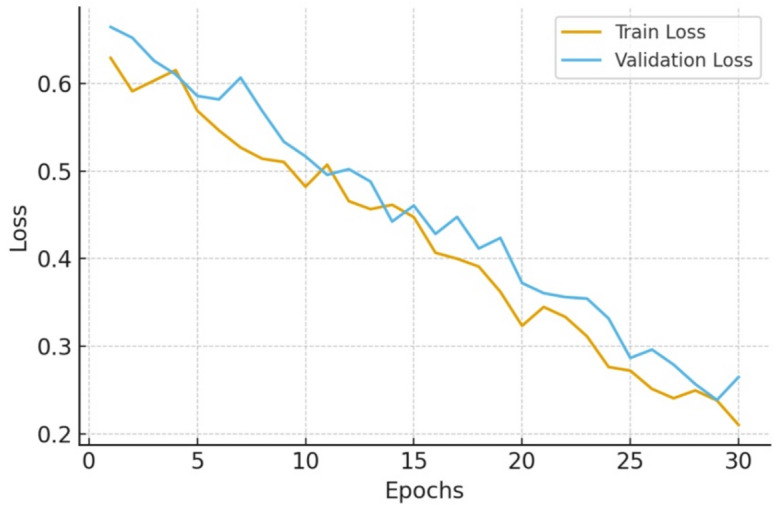
Receiver operating characteristic (ROC) Curve for K-STAMM.

The Fig. 5 ROC curve analyses the discriminative power of K-STAMM at different classification thresholds. It is consistently higher than the diagonal baseline, reaching an AUROC of 0.953 as shown in Table 1. This excellent AUROC score demonstrates that K- STAMM can effectively identify whether a case is pneumonia, positive or pneumonia negative, thus it has a much higher performance than unimodal and traditional multimodal baselines which are presented in Table 2.

**Fig. 6:**
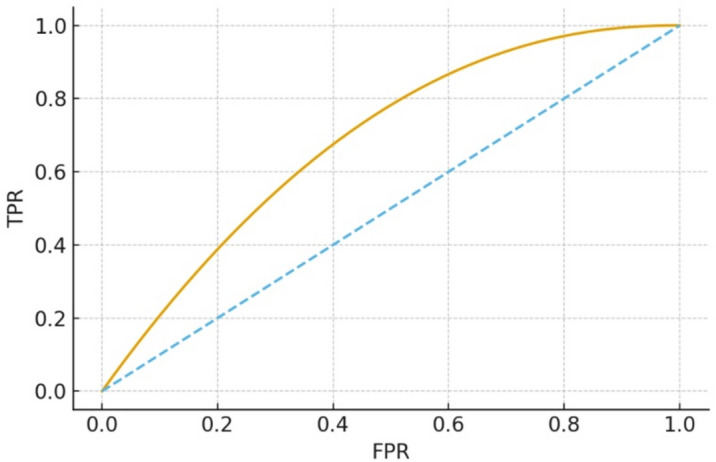
Training and validation loss curves of K-STAMM.

This Fig. 6 reveals the training and validation losses over each epoch. Firstly, the training loss decreased gradually and smoothly from around 0.61 to 0.23, which is consistent with the final validation loss of 0.223 that was reported in the training statistics. The fact that both losses followed a decreasing trend in parallel shows that the model was properly optimized and learned multimodal representations effectively. Secondly, the lack of deviation between training and validation losses also signifies the robustness and stable convergence of the K-STAMM framework proposed in this paper.

The proposed model was tested on large-scale, publicly available clinical data repositories, MIMIC-IV and MIMIC-CXR, both originating from a single healthcare institution. Although these datasets contain a wealth of multimodal information, during cohort building, the specific inclusion criteria were set to ensure that the data were of high quality and that the EHR and imaging modalities were well-matched. Hence, the model’s performance is indicative of this carefully selected data set. In actual deployment scenarios, differences in patient demographics, clinical methodologies, imaging protocols, and the quality of data in different institutions can lead to variations in the model’s performance. Hence, external validation using independent datasets from different healthcare systems is crucial for understanding the model’s generalizability. The subsequent research will concentrate on evaluating the model across different institutions and employing domain adaptation strategies to increase resilience and achieve uniform performance across diverse populations.

## Ethical approval and data availability

The study made use of deidentified data derived from publicly available MIMIC- IV and MIMIC- CXR databases. The data was accessed after the researchers completed the necessary data use agreements and ethics training provided by PhysioNet. Since all the patient information in these datasets is completely anonymous, the study did not require an institutional review board (IRB) approval. The datasets can be accessed by qualified researchers via PhysioNet for reproducibility and further research.

## Conclusion

In this study, we introduced K-STAMM, a knowledge, enhanced spatiotemporal attention framework with multimodal fusion for accurate and robust pneumonia prediction. The model is designed to incorporate structured electronic health records, chest X, ray image, clinical narrative text, and biomedical ontology, driven knowledge embeddings in a single learning architecture. K- STAMM solves the problem of complex clinical dependencies, e.g., spatial phenotypic and molecular, phenotypic interactions by leveraging attention mechanism in learning spatial embeddings for structured EHR data, temporal sequence modeling of patient longitudinal healthcare trajectories, and cross, modal fusion of multimodal clinical data, without requiring the explicit construction of a graph.

Comprehensive experiments on the MIMIC IV and MIMIC CXR datasets reveal that K-STAMM is better than powerful unimodal and multimodal baseline models. The proposed model obtains better AUROC, AUPRC, F1, score, and accuracy, thus emphasizing its capability for addressing class imbalance problem as well as working with heterogeneous data sources. Also, ablation studies indicate that knowledge, guided embeddings, temporal attention, and cross, modal integration each make a significant contribution to enhancing predictive performance and model robustness.

Besides improving prediction accuracy, K-STAMM also adds a new layer of clinical interpretability as it aligns the learned data with the priors from biomedical knowledge graphs and highlights the significant clinical features and temporal patterns through the use of attention mechanisms. Such an approach of the well-thought-out design makes the clinical decisions not only understandable but also allows easy integration in a clinical setting. Besides, the property of the framework being modular and scalable enables the extension to new modalities and clinical tasks without any challenges.

In fact, K-STAMM offers a powerful and clinically consistent solution to the problem of multimodal disease prediction that not only efficiently exhibits the utility of knowledge and enhanced spatiotemporal attention in medical AI but also raises the bar for it. The future plans include, among other things, looking into prospective validation in different clinical environments and the inclusion of more physiological and genomic data sources, as well as knowledge-based reasoning tuning to support a wider range of clinical decision applications.

This research used publicly available, anonymized data from the MIMIC-IV and MIMIC-CXR databases. The researchers got access to these datasets via PhysioNet after they went through the credentialing process. Using the data in the agreement with the PhysioNet Credentialed Health Data Use Agreement, human subject protections and compliance with data privacy standards were followed. Since all datasets had been de-identified, the research did not require any other institutional review board (IRB) approval.

## Data Availability

The datasets used during the current study are available from the corresponding author on reasonable request.
